# Liquid Crystalline Behavior and Related Properties of Colloidal Systems of Inorganic Oxide Nanosheets

**DOI:** 10.3390/ma2041734

**Published:** 2009-10-29

**Authors:** Teruyuki Nakato, Nobuyoshi Miyamoto

**Affiliations:** 1 Division of Bio-Applications and Systems Engineering (BASE), Institute of Symbiotic Science and Technology, Tokyo University of Agriculture and Technology, 2-24-16 Naka-cho, Koganei-shi, Tokyo 184-8588, Japan; 2 Department of Life, Environment, and Materials Science, Faculty of Engineering, Fukuoka Institute of Technology, 3-30-1 Wajiro-higashi, Higashi-ku, Fukuoka-shi, Fukuoka 811-0295, Japan; E-Mail: miyamoto@fit.ac.jp

**Keywords:** layered compound, inorganic nanosheet, colloid, liquid crystal, sol–gel transition, clay, semiconductor photocatalyst

## Abstract

Inorganic layered crystals exemplified by clay minerals can be exfoliated in solvents to form colloidal dispersions of extremely thin inorganic layers that are called nanosheets. The obtained “nanosheet colloids” form lyotropic liquid crystals because of the highly anisotropic shape of the nanosheets. This system is a rare example of liquid crystals consisting of inorganic crystalline mesogens. Nanosheet colloids of photocatalytically active semiconducting oxides can exhibit unusual photoresponses that are not observed for organic liquid crystals. This review summarizes experimental work on the phase behavior of the nanosheet colloids as well as photochemical reactions observed in the clay and semiconducting nanosheets system.

## 1. Introduction

**Figure 1:**
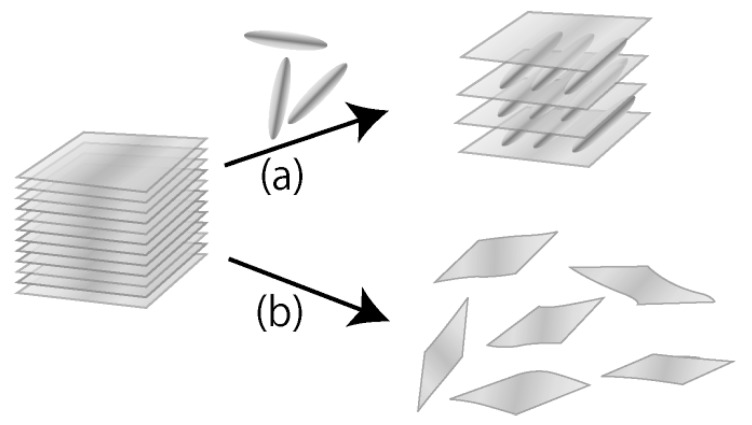
Schematic representation of: (a) intercalation and (b) exfoliation.

Inorganic layered compounds are crystalline solids built up from stacked inorganic sheets of around 1-nm thickness. Many layered compounds are known to accommodate guest molecules into their interlayer spaces, where the incorporated molecules are arranged in unusual manners reflecting two-dimensionally restricted interlayer arrays, as schematically shown in Figure 1a [1,2]. This inclusion phenomenon is called intercalation. The intercalated molecules often exhibit unusual properties that are not observed in homogeneous states such as solutions [3,4,5,6,7,8]. When the layered compounds are electrochemically and/or photochemically active, electronic and energetic communication can occur between the inorganic layers and the intercalated guest species upon electro- and/or photo-stimuli [9,10,11,12]. Since the layered compounds are rigid solids, they are classified into hard matter, being far from soft matter exemplified by liquid crystals. Thus, intercalation is regarded as a method to “immobilize” the guest molecules between the crystalline inorganic layers. Consequently, materials chemistry of inorganic layered compounds has been recognized as an area of solid state chemistry for a long time.

Recently, another type of interlayer reaction of inorganic layered compounds, which is called exfoliation, has attracted great attention, as illustrated in Figure 1b [13,14,15,16]. By exfoliation, individual crystalline sheets of layered compounds are liberated from the stacked state. Since exfoliation is usually carried out in solution systems, the exfoliated “nanosheets” are yielded in colloidally dispersed state in most cases. However, researches on exfoliation of layered crystals are concentrated on restacking of the dispersed nanosheets. Restacking is regarded as a bottom-up re-building process of layered solids. The exfoliation–restacking route has been utilized to incorporate various molecular and cluster species which often cannot be accommodated in the interlayer spaces by direct reactions of layered solids with the guest species. Thus, exfoliation (and subsequent restacking) has been recognized as a method alternative to intercalation for immobilizing functional molecules in the interlayer spaces of stacked inorganic nanosheets. In contrast, the colloidally dispersed state of the exfoliated nanosheets has attracted little attention except for the colloids of clay minerals.

We have investigated colloidal states of crystalline nanosheets prepared by exfoliation of inorganic oxides in recent years, and found that the “nanosheet colloids” exhibit various unusual properties which are not observed in layered solids such as parent layered crystals and exfoliated–restacked assemblies of nanosheets. Our studies demonstrate that the nanosheet colloids are a kind of soft matter whose structural motif is flexible and relatively loose ordering of the colloidal nanosheets based on weak interactions between the inorganic structural units. Moreover, if we use nanosheets of photocatalytically active wide band-gap semiconductors, we are able to cause photochemical reactions initiated by photoexcitation of the semiconducting nanosheets and the reactions are controlled by colloidal conditions of the system. In this review, we summarize our researches and related works of the nanosheets colloids of layered clay minerals and semiconducting oxides. It covers experimental results of liquid crystal formation and physical gelation as well as photochemical electron transfer reactions observed in the nanosheet colloids.

## 2. Nanosheet Colloid as a Colloidal System of Anisotropic Particles

Nanosheet colloids prepared by exfoliation of layered crystals are members of the colloids of anisotropic particles such as rods and plates. We call such colloidal systems “anisotropic colloids” hereafter. The anisotropic particles have larger excluded volumes than spherical ones, and show specific phase behavior in the colloidal states reflecting the shape of particles. Also, the colloids anisotropically respond to external fields to exhibit various characteristic behaviors. The exfoliated nanosheets are plate-like particles with very high aspect ratio because they have thickness of around 1 nm and lateral dimension up to several micrometers. We describe below some fundamental characteristic properties of the nanosheet colloids represented by liquid crystalline phase behavior.

## 3. Liquid Crystallinity of the Anisotropic Colloids

### 3.1. Theoretical backbone

Liquid crystalline phase behavior is one of the most important properties characteristic to anisotropic colloid. Liquid crystallinity of the colloids means that the particles are dispersed in the solvent with orientational order. A colloid that is isotropic at low particle concentrations transits to a biphasic mixture of isotropic and nematic phases, and finally to a single nematic phase as a result of entropic driving force; thus, the liquid crystalline behavior is lyotropic.

**Figure 2:**
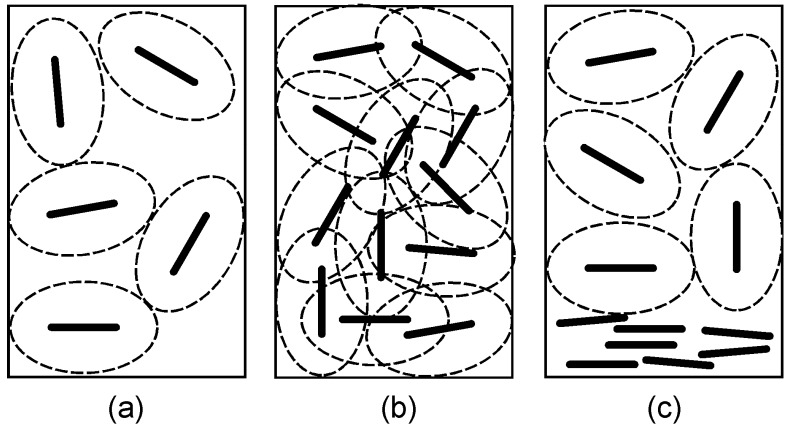
Schematic representation of the isotropic–nematic phase transition in an anisotropic colloid. The colloidal particles (represented by a rod) are randomly dispersed to form an isotropic phase at a low concentration (a). However, such an orientation becomes impossible by overlap of excluded volume (represented by an ellipse surrounded with dashed line) of the particles at a high concentration (b). When a part of the particles are orientationally ordered to form a liquid crystalline phase, excluded volume of the residual particles is recovered (c).

This behavior is basically explained by the Onsager theory based on the excluded volume effect [17,18]. Since anisotropic particles have large excluded volumes, freedom of particle movement is restricted at high particle concentrations due to overlap of their excluded volumes to induce entropic loss. Such a system can be thermodynamically stabilized if part of the particles are orientationally ordered because total entropy of the system, which is composed of the arranged particles retaining translational freedom and the residual ones whose excluded volume is recovered, becomes larger than that assumed for the all-isotropic system with the restricted excluded volume. These situations are schematically shown in Figure 2. The Onsager theory gives a fundamental theoretical framework to the phase behavior of anisotropic colloid, and there are many studies on improvement of the theory and simulation experiments [18,19,20,21,22,23,24,25].

### 3.2. Historical overview

As to experimental studies of anisotropic colloid, liquid crystalline behavior of the colloids of virus rods [26], V_2_O_5_ rods [27], and clay plates [28] had been reported in early 20th century; the Onsager theory was established on the basis of these observations. However, relatively few experimental investigations had been conducted successively after these historical works, and many of the studies were related to the colloids of rods [29,30,31,32,33,34,35,36,37,38,39,40,41,42]; thus, the anisotropic colloids, in particular colloids of plate-like particles, had been “forgotten systems” for a long time. This situation has been changed from the end of the past century, since two groups, Lekkerkerker *et al*. [43] and Davidson *et al*. [44], began systematic investigations. Liquid crystalline phase behavior of boehmite [45,46,47] and goethite [48,49,50,51,52,53] rods, and gibbsite [43,54,55,56,57,58,59,60,61,62,63,64] and nickel hydroxide [65] plates has been studied and discussed in relation to the theoretical considerations. Recently, liquid crystallinity of the colloids of functional rod-like particles exemplified by DNA [66,67,68], metal [69,70] and semiconductor [71,72,73,74,75,76] nanorods, and carbon nanotubes [77,78,79,80,81] has attracted attention from a viewpoint of applying these nano-objects to advanced materials.

### 3.3. Clay colloids

**Figure 3:**
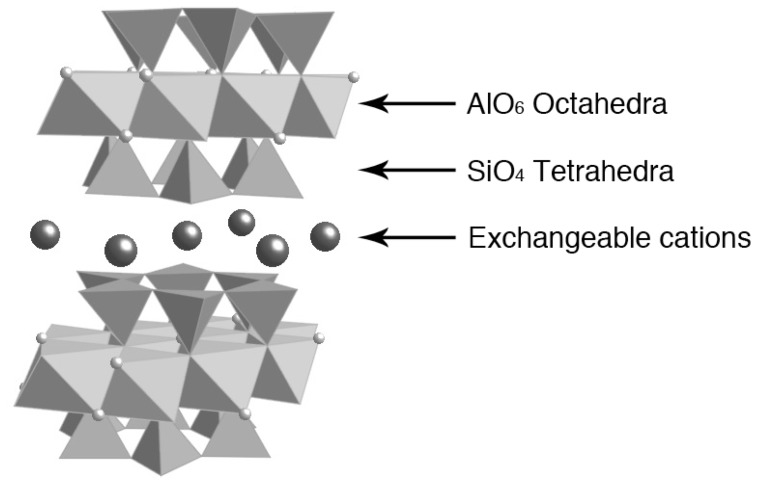
Crystal structure of smectite-type clay, montmoriilonite.

Among various layered materials, smectite-type clay minerals are most widely known and have long been investigated. Figure 3 represents the schematic structure of montmorillonite, a typical smectite-type clay mineral [2,82]. The aluminosilicate layer of montmorillonite is composed of two corner-shared SiO_4_ tetrahedral sheets sandwiching an edge-shared Al(O,OH)_6_ octahedral sheet. The clay layer is characterized by permanent negative charge due to isomorphous substitution of Al^3+^ atoms of the octahedra for lower valent cations (Mg^2+^). To compensate the negative charge, exchangeable cations (Na^+^) are present in the interlayer spaces. The interlayer cations are easily solvated by polar solvents, allowing the smectite-type clays to undergo exfoliation. In addition to the permanent charge, pH-dependent charge is present on the edges of clay layer; Al–OH (or Si–OH) groups at the edges are present as different states depending on pH of the solution: Al–OH_2_^+^ (at low pH), Al–OH (at medium pH), and Al–O^–^ (at high pH).

Other than montmorillonite, there are several kinds of smectite-type clay minerals (e.g., saponite, hectorite, beidelite, and nontronite), which differ in chemical composition and the type of isomorphous substitution. Most of these clay minerals occur naturally in earth soils, whereas the composition, isomorphous substitution, impurity level, and crystallinity of the clays differ depending on the production areas. Synthetic clay minerals, which are suitable for chemical experiments due to lower impurity content, are also available.

Liquid crystalline behavior of clay colloid was observed at first for bentonite clay (a natural clay mainly composed of montmorillonite) by Langmuir in 1938 as mentioned above [28]; nevertheless, the next unambiguous evidence for the liquid crystallinity of clay colloid has not been reported for a long time. In 1996, Gabriel *et al*. recalled the liquid crystallinity of clay colloid, and presented a phase diagram of colloidal systems of bentonite and hectorite [44]. Their result triggered studies of the phase behavior of clay colloid [83,84,85,86,87,88,89,90,91,92,93,94,95], but the discussion has not reached a common conclusion. This is probably due to gelation of clay colloid, the behavior which usually competes with the isotropic–nematic phase transition. In the gelled clay colloids, spontaneous orientation of clay platelets reflecting liquid crystallinity is difficult to be obtained because shear-induced orientation, which is unavoidable in handling of the samples (such as mechanical stirring of the colloids), is not relaxed in a short period to arrest the particles in a non-equilibrium state [44,96]. In addition, clay colloids have been thought for a long time to form “house-of-cards” structures where the clay platelets are assumed to be distributed randomly [82]. This idea does not easily accept the orientational ordering of the clay particles characteristic to the liquid crystalline phase.

**Figure 4:**
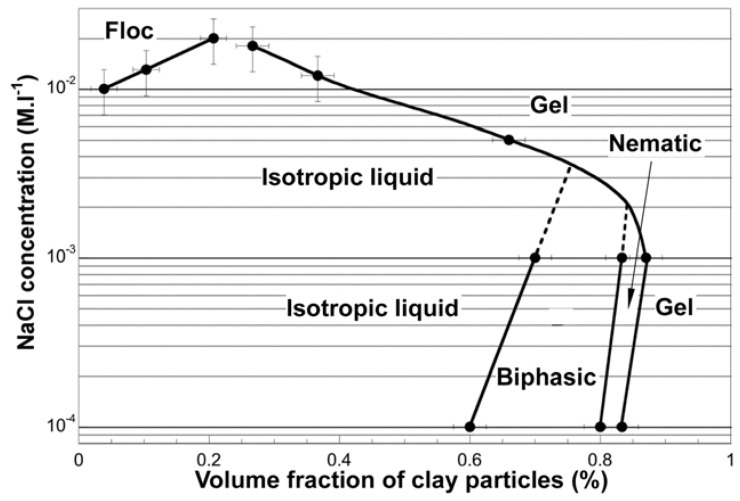
Phase diagram of an aqueous colloid of nontronite clay [96]. Copyright (2006) National Academy of Sciences, USA.

However, Michot *et al*. have recently succeeded in observing the phase behavior with separation of the isotropic–nematic transition and gelation by the use of nontronite clay [96,97]. Figure 4 shows a typical phase diagram. It clearly shows lyotropic phase transition from isotropic to biphasic and finally to nematic with increasing the particle concentration. This result and some other results described below demonstrate that the lyotropic liquid crystalline behavior is a common property of the nanosheet colloids prepared from layered compounds. The diagram also indicates an electrolyte effect that is aggregation of the platelets at high electrolyte concentrations by screening of the electric double layer; hence, stable colloidal dispersions and thus the liquid crystals are observed at appropriately low electrolyte concentrations.

### 3.4. Layered phosphate

In 2001, Gabriel *et al*. reported phase behavior of nanosheet colloid prepared from layered phosphate H_3_Sb_3_P_2_O_14_ [98], the work which has sparred extension of the liquid crystalline behavior of inorganic nanosheets to layered compounds other than clays. The layered phosphate spontaneously swell in water, similarly to the clay minerals, to form colloidally dispersed nanosheets. Phase behavior of H_3_Sb_3_P_2_O_14_ nanosheet colloid is basically the same as that of clay colloid as characterized by the isotropic–biphasic–liquid crystalline transition. Differences are that the liquid crystalline phase is lamellar and that the phase transition from isotropic to biphasic is observed at a much lower nanosheet concentration than that of the clay system. The latter is ascribed to large lateral size of the nanosheets as described below.

### 3.5. Layered niobates and titanates

**Figure 5:**
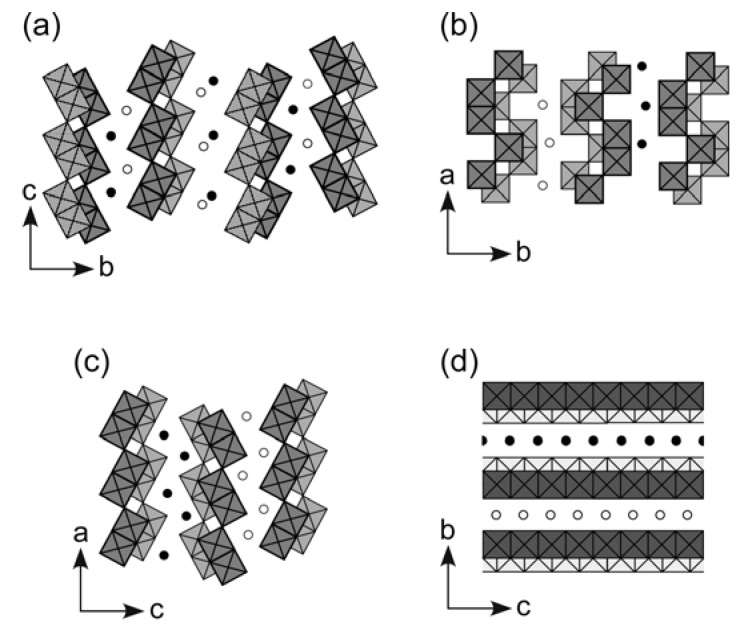
Crystal structure of layered niobate and titanates that have intercalating reactivity and semiconductor photocatalytic activity: (a) K_4_Nb_6_O_17_, (b) HNb_3_O_8_, (c) HTiNbO_5_, and (d) H_1.07_Ti_1.73_O_4_. Squares and circles indicate NbO_6_ or TiO_6_ octahedra that form oxide layers and interlayer exchangeable cations (K^+^ or H^+^), respectively.

**Figure 6:**
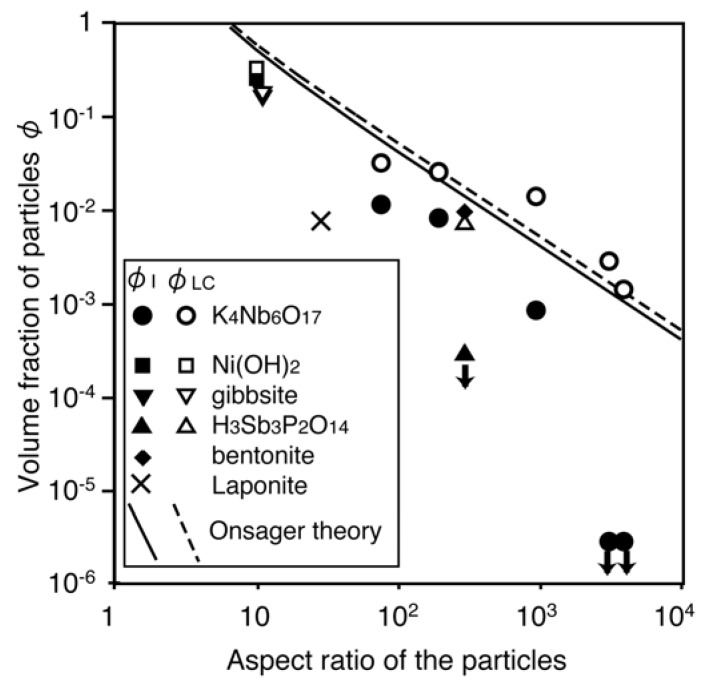
Relationship between aspect (diameter to thickness) ratio of colloidal disks and the transition concentrations *φ*_I_ (isotropic–biphasic) and *φ*_LC_ (biphasic–liquid crystalline) observed for the niobate nanosheet colloid with those in the literatures [100]. Solid and dashed lines represent the theoretical values numerically calculated based on the Onsager theory. The down arrows indicate that the transition concentration was not determined but is lower than the value indicated by the symbols. Reprinted with permission from Ref. [100]. Copyright 2004 American Chemical Society.

We reported lyotropic liquid crystalline behavior of nanosheet colloid of layered niobate K_4_Nb_6_O_17_ for the first time in 2002 [99]. The layered structure of K_4_Nb_6_O_17_ consists of corner- and edge-shared NbO_6_ octahedra and interlayer exchangeable K^+^ ions, as shown in Figure 5a. Exfoliation is achieved by exchange of the interlayer cations for propylammonium ions that are introduced as an exfoliating reagent. Since we prepared large nanosheets by exfoliating large crystals of the niobate, the obtained liquid crystal showed gravity-induced macroscopic orientation. We also prepared the niobate nanosheet colloids with controlled nanosheet sizes by exfoliation of single crystals and subsequent ultrasonic irradiation [100]. Figure 6 shows phase transition concentrations (isotropic–biphasic and biphasic–liquid crystalline) of the niobate nanosheet colloids as a function of the aspect ratio (average lateral dimension of the nanosheets divided by their thickness), comparing with those found in previous experimental studies of the colloids of plate-like particles and in a theoretical study reporting numerical solution of the Onsager theory [101]. The phase transition concentrations decrease with increasing the aspect ratio of the nanosheets, and basically agree with the theoretical values. This diagram explains that the phase behavior of niobate nanosheet colloid is basically compatible to the theoretical regime of Onsager. The nanosheets with larger aspect ratio (namely, larger lateral size) have larger excluded volumes, and thus lead to lower phase transition concentrations.

We also confirmed that some other layered niobates and titanates—HNb_3_O_8_, HTiNbO_5_, and H_1.07_Ti_1.73_O_4_—form liquid crystalline phases [102,103]. These protonic compounds and K_4_Nb_6_O_17_ have similar structures and physicochemical properties; they are structurally related to each other, as shown in Figure 5b–d, and exhibit semiconductor photocatalysis. The protonic layered niobates and titanates are exfoliated in water by using tetrabutylammonium ions as the exfoliating reagent to form nanosheet colloids. The obtained nanosheet colloids show liquid crystalline behavior essentially the same as that of K_4_Nb_6_O_17_. However, phase transition concentrations vary with the exfoliated species at a similar lateral dimension of the nanosheets. Colloids of the protonic niobates and titanates all show higher isotropic–biphasic transition concentrations than K_4_Nb_6_O_17_. Thus, liquid crystalline phases of these protonic oxides are less stable than K_4_Nb_6_O_17_. The less stable liquid crystalline phases are probably related to flexibility of the nanosheets and incomplete exfoliation for these layered niobates and titanates. The layered crystals other than K_4_Nb_6_O_17_ are exfoliated into monolayer-type nanosheets, whereas K_4_Nb_6_O_17_ forms bilayer-type nanosheets because of its characteristic layered structure [104,105]. Therefore, the nanosheets of these oxides should be more flexible than those of K_4_Nb_6_O_17_; the flexibility of the nanosheets can reduce the effective lateral size (persistence length) of the nanosheets. Also, incomplete exfoliation would lead to increase in the nanosheet thickness (decrease in the aspect ratio) and decrease of number density of the nanosheets (decrease in the total excluded volume). These situations lead to decrease in the total effective excluded volume, resulting in the destabilization of liquid crystalline phases.

Layered niobate K_4_Nb_6_O_17_ can become dispersed in nonpolar organic solvents after modification of the sheet surfaces with hydrophobic species [106]. The surface modification is achieved by covalent attachment of octadecyltrimethylsilane molecules onto the niobate layers through pre-intercalation of bulky organoammonium ions into the interlayer space of K_4_Nb_6_O_17_ and subsequent silane coupling reaction with the organosilane species. The silylated niobate forms stable colloidal dispersion in chloroform, where stacking of the niobate layers is lost as evidenced by X-ray diffraction. The obtained chloroform suspension exhibits lyotropic liquid crystallinity.

### 3.6. Layered hydroxides

Liquid crystalline behavior of colloidal dispersions of layered double hydroxides (LDH) [Mg_1–*x*_Al*_x_*(OH)_2_](A*^n^*^–^)*_x_*_/*n*_·*m*H_2_O (A*^n^*^–^: inorganic or organic interlayer anions compensating the positive charge of the [Mg_1–*x*_Al*_x_*(OH)_2_]*^x^*^+^ layers) has recently been discovered [107,108,109]. The LDH crystallites are dispersed in water as platy particles. The phase transition occurs at relatively high particle concentrations (>10wt%) compared with those of the colloids of exfoliated nanosheets described above. This is explained by that the LDH crystals are not exfoliated to the nanosheets but present as thick platelets (thickness of several nanometers) whose excluded volume is not as large as that of exfoliated nanosheets because of relatively low anisotropy. The obtained liquid crystalline phase is usually assigned to nematic but to lamellar in some cases depending on the surface charge density of the LDH platelets, suggesting contribution of electrostatic interactions between the colloidal particles to the phase behavior.

Layered copper hydroxycarboxylates Cu_2_(OH)_4–*x*_(C*_n_*H_2*n*+1_COO)*_x_* (*n* = 7–12) form stable colloidal dispersions in toluene and exhibit liquid crystallinity induced by lyotropically as well as thermotropically [110]. The layered compounds are suspended as platy particles with thickness of several tens of nanometers. The obtained colloids with concentrations of several weight percents are separated into the particle-suspended and pure solvent phases, and the former phase show lyotropic liquid crystallinity. However, the liquid crystalline phase is observed only below 54–83 °C depending on carbon number (*n*) of the alkyl chains; the colloids form transparent isotropic phases over the phase transition temperature.

### 3.7. Phase separation in multi-component anisotropic colloids

Anisotropic colloids composed of more than two kinds of particles with different shape show richer phase behavior than single-component colloids. This is theoretically predicted and experimentally confirmed. For example, three-phase coexistence (one isotropic and two nematic phases) is proposed for a double-component colloidal system of rods and discs by calculation [111], and a corresponding experimental system composed of boehmite (AlOOH) rods and gibbstite (Al(OH)_3_) plates shows five-phase coexistence (isotropic, rod-dominant nematic, plate-dominant nematic, unidentified nematic, and columnar phases) [112,113].

These results indicate that the phase separation of multi-component anisotropic colloid induces fractionation of the dispersed particles based on their shape. The fractioned components composed of the morphologically similar particles form their own liquid crystalline domains that are structurally different from each other reflecting the shape of particles. Structure of the multi-phase coexisting colloids can also be recognized hierarchically; individual particles form a liquid crystalline domain and the domains are collected to give a higher-order structure of the colloids.

We have prepared a binary anisotropic colloid composed of two kinds of nanosheets prepared by exfoliation of montmorillonite clay and layered niobate [114]. Optical microscope observations indicate that the obtained colloid, called clay–niobate colloid hereafter, is apparently homogeneous in macroscopic (~sub-mm) scale to show liquid crystallinity. However, spectroscopic observations by using an organic dye as a probe evidenced demixing of the clay and niobate nanosheets at microscopic level. Visible spectroscopy of the clay–niobate colloid added by a cationic organic dye indicates that the dye molecules are selectively adsorbed on the clay nanosheets because of hydrophobicity of the clay surface, and that the dye adsorbed on the clay platelets does not show spectral dichroism. These facts mean that the clay particles are isotropically dispersed in the clay–niobate colloid; thus, the clay nanosheets do not contribute to the liquid crystallinity of the colloid. Consequently, in the clay–niobate colloid, the clay and niobate nanosheets are phase-separated to form isotropic and liquid crystalline domains, respectively.

Other examples of multi-component colloid related to inorganic nanosheets are found for clay nanosheets and LDH platelets. A binary colloid system composed of clay nanosheets and Fe_2_O_3_ spherical nanoparticles shows phase separation of the two colloidal particles at a microscopic level similarly to the clay–niobate colloid although both of the particles are isotropically dispersed [115]. A system of LDH platelets and poly(ethylene glycol) polymers show multi-phase coexistence including plural liquid crystalline phases [116]. A recent paper indicates phase separation of an aqueous colloidal dispersion of manganate nanorods added by graphene oxide nanosheets, which enrich the nanorods at the air-water interface and thus induce orientational ordering of the concentrated rods at the interface [117]. On the other hand, a colloidal mixture of clay nanosheets and surface-modified carbon nanotubes has been reported to form a homogeneous suspension [118]. In this system, the clay nanosheets have been assumed to adhere to the surface of the carbon nanotubes. This alters the surface property of carbon nanotubes to hydrophilic, and thus increases their dispersibility.

## 4. Ordering of the Nanosheets by External Forces

Orientation of the colloidally dispersed anisotropic particles containing nanosheets can be anisotropically arranged in macroscopic scale by applying external fields such as shear, electric, and magnetic fields because of the anisotropic interactions of the particles and fields. The anisotropic orientation is optically detected by characteristic birefringence.

Even below the critical concentration of isotropic-to-liquid crystalline phase transition, birefiringence due to nanosheet orientation is observed when the nanosheets are under shear flow; this phenomenon is called flow-birefringence. An isotropic niobate nanosheet colloid in a test tube shows clear flow-birefringence when the tube is rotated [100]. This birefringence is temporal and gradually disappears after termination of the rotation. The relaxation time becomes longer as the concentration of the isotropic colloid approaches to the critical concentration of the isotropic–biphasic transition. Flow-birefringence is due to temporal ordering of the anisotropic particles by shear flow. Structural investigation by small-angle scattering techniques showed that clay nanosheets (hectorite and montmorillonite) under shear are preferentially aligned in the direction of flow under low shear, while that the oriented structure is broken under very high shear [119,120,121]. Shear-induced orientation has also been reported for the colloidal mixtures of clay nanosheet and polymers such as hectorite–poly(ethylene oxide) [122,123,124,125] and montmorillonite–polybutadiene systems [126]. Temporal birefrin-gence is also induced by electric field as known as electro-birefringence. Electro-birefringence of clay colloids was discovered in early 1960s, and have continuously been studied by a few research groups [127,128,129,130,131,132,133,134,135].

Liquid crystal phases of magnetically active nanosheets are readily aligned macroscopically by a moderate magnetic field. V_2_O_5_ ribbons form a single oriented domain by applying a magnetic field of 1 T for 5 min or 0.3 T for 2 h [136,137,138]. Nanosheets of nontronite clay which has ferric ions in the AlO_6_ octahedra are also oriented by a magnetic field of 1 T [96]. The macroscopic alignment of the liquid crystalline phases are applicable for the synthesis of ordered porous monoliths [138] and anisotropic media for NMR measurement of nonlabeled biomolecules [137].

Diamagnetic nanosheets can be magnetically aligned if a strong magnetic field is applied for a long time, as reported for the liquid crystalline phase of H_3_Sb_3_P_2_O_14_ [98] and a birefringent gel phase of fluorohectorite [139]; magnetic fields of 18.7 T for 10 min and 2 T for 36 h are required for the phosphate and clay, respectively. Niobate nanocrystals and nanoscrolls prepared by exfoliation of K_4_Nb_6_O_17_ can be aligned without surface modification by applying a strong magnetic field of ~12 T [140]. However, alignment of diamagnetic nanosheets with a magnetic field is enhanced by modifying the colloidal nanosheets with magnetic species. Kim *et al*. have reported alignmnent of exfoliated nanosheets prepared from perovskite-type niobate HCa_2_Nb_3_O_10_ by attaching magnetite nanoparticles through linker molecules covalently bound to the niobate surfaces [141].

**Figure 7:**
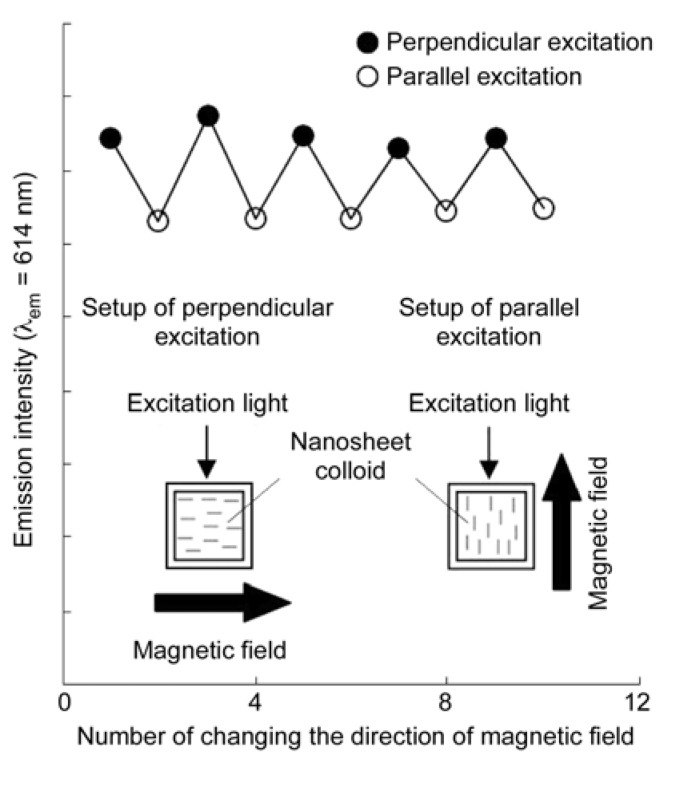
Modulation of emission intensity of a Gd_1.4_Eu_0.6_Ti_3_O_10_ nanosheet colloid with applying a perpendicular or parallel magnetic field applied (λ_ex_: 250 nm and λ_em_: 614 nm) [142]. Reprinted with permission from Ref. [142]. Copyright 2007 American Chemical Society.

Magnetic alignment of nanosheet has been applied to control optical properties of nanosheet colloids. Ida et al. have investigated photoluminescence of magnetically aligned nanosheet colloids of perovskite-type layered oxides K_2_Ln_2_Ti_3_O_10_, K_2_Ln_2_Nb_2_O_7_, and Rb_2_Ln_2_Ta_2_O_7_ (Ln = Gd, Eu, Tb) [142]. They have indicted that luminescence intensity due to the rare earth ions embedded in the oxide layers depends on the angle between the direction of incident beam for excitation and that of magnetic field to show dichroic behavior. Emission intensity becomes a maximum when the excitation beam is perpendicular to the magnetic field, and is reversibly controlled by alteration of the direction of external field, as shown in Figure 7.

## 5. Physical Gelation of the Nanosheet Colloids

### 5.1. Gelation of clay colloid

Colloids of smectite-type clay minerals are gelled at enough high concentrations of the clay. This property has been one of central interests of clay colloid science from the viewpoints of condensed matter physics and many practical applications for paints, cosmetics, drilling fluids, etc. Sol–gel phase transitions of clay colloids of Laponite (synthetic hectorite) and montmorillonite have been investigated as functions of clay concentration and ionic strength of added indifferent electrolytes [44,143]. The sol–gel transition is observed at a clay concentration of 1–2wt% and this critical concentration depends on the ionic strength. Clay particles are flocculated at very high ionic strength as is the general behavior of colloid systems. However, the phenomenon is complicated and problematic. Since the sol–gel transition is very slow, it continues for a time scale of years. Insufficient washing of the clay particles often carries over electrolytes to the system as impurities to give incorrect phase diagrams. Although numerous studies of clay colloid have been carried out, “there is not yet a clear and convincing explanation” [86,92].

### 5.2. Consideration by classical theorems of viscosity

A classical description of relative viscosity *η*_r_ of a colloidal system is given by the Einstein equation:

*η*_r_ = 1 + *Kφ* (1)

where *K* is the constant, being 2.5 for spheres and >2.5 for anisotropic particles, and *φ* is the colloid concentration. This equation rationalizes the viscous nature of colloidal dispersions of anisotropic clay particles and larger viscosity for more concentrated clay colloids. However, it does not predict the rheological behavior, in particular the concentration-induced gelation of clay colloid.

We may also recall the general relationship between the viscosity and the molecular weight of a dispersoid. Limiting viscosity [*η*] of a chain polymer solution is expressed by the Mark–Houwink–Sakurada equation:

[*η*] = *kM^a^* (2)

where *M* is the molecular weight of polymer and *k* and *a* are constants. If we assume the nanosheets as inorganic polymers, we expect larger viscosity for the colloids of nanosheets with larger sizes. Actually, the niobate nanosheet colloid with the average lateral nanosheet size of 7.8 μm shows over 50-times larger kinematic viscosity than that with the nanosheet size of 0.15 μm at the volume fraction of 0.7% [100].

However, clay colloid shows different behavior. Colloids of Laponite are very viscous and give a sol–gel transition concentration (below 0.5% of volume fraction) much lower than that of the niobate system [44,143] although the lateral size of Laponite platelets (around 25 nm [144]) is much smaller than the niobate nanosheets. Clay colloids of montmorillonite and nontronite minerals with controlled nanosheet sizes show an abnormal trend: larger viscosities for smaller nanosheets [91,97,145]. For example, montmorillonite colloids with average nanosheet sizes of 410 and 75 nm are gelled at 20 and 14 g L^–1^, respectively. Hence, the simple classical descriptions are not enough for understanding the gelation of clay colloid.

### 5.3. Structural model of gelled clay colloids

Two incompatible structures—“house-of-cards” and glass-like structures—have been proposed for the structure of gelled clay colloids [92]. The “house-of-cards” structure is a classical model that assumes formation of a large network structures by electrostatic attraction between the negatively charged surfaces and positively charged edges of the clay platelets [82,92,121,146,147]. The network structure has been supported by small-angle scattering measurements [121,143,148,149,150,151]. The obtained scattering profiles are assigned to fractal-like superstructures. However, positive charges of the nanosheet edges are effective only at low pHs, where the Al–OH or Si–OH groups are protonated, as verified by ζ-potential measurements [152]. Because the sol–gel transition of clay colloid is observed even at higher pHs, the formation of “house-of-cards” structure cannot be the only cause of the sol–gel transition. Surface-to-surface interactions should contribute to the gel structure.

The glass-like structure assumes electrostatic repulsion between the anionic clay platelets, the repulsion which leads to arrest of the clay particles [95,143,153]. Mourchid *et al*. have reported that the osmotic pressure of Laponite colloid is always positive and it decreases with increasing ionic strength, confirming that the repulsive interaction dominates in this system [83,143,148]. Moreover, Michot *et al*. have recently investigated nontronite colloid with varied nanosheet sizes, nanosheet concentrations, and electrolyte concentrations. They have shown that the plots of yield stress and viscosity against nanosheet concentration are expressed by a simple theoretical master curve after correction of the nanosheet concentration for Debye screening length and excluded volume of the platelets, and concluded that only repulsive interactions are present in the system [91,97,145]. However, the presence of long-range structures found in small-angle scattering experiments in the colloids [121,123,148,149,150,151] are not compatible with this repulsion-only model as Mourchid *et al.* themselves have questioned [143].

Recent studies propose that both the types of the gel phases are present in a phase diagram of clay colloid. Tanaka *et al*. have proposed that the apparent gelation of clay colloids is classified into liquid–glass and sol–gel transitions [90,153], which give three different gel phases: (i) repulsive glass (Wigner glass) stabilized only by electric repulsion, (ii) attractive glass stabilized by both attractive and repulsive intereactions, and (iii) attractive gel with large network structures formed through electrostatic attractive interactions. The phase diagram of montmorillonite colloid obtained by Abend and Lagaly is consistent with this theory [154]. The rheological behavior of montmorillonite colloid added by electrolytes supports this model [155,156]. Viscosity of the colloid decreases by adding small amounts of electrolyte, and then steeply increases by adding larger amounts of them. It is explained that the electrostatic repulsion between the clay nanosheets, the interaction which stabilizes the repulsive gel, is screened when small amounts of electrolyte are added, while that the attractive interactions between the clay particles dominate at higher salt concentrations to generate the network structure of the attractive gel. When an acid is added as the electrolyte, the changes in rheological behavior occur at lower concentrations due to more positive charges at nanosheet edges at lower pHs.

**Figure 8:**
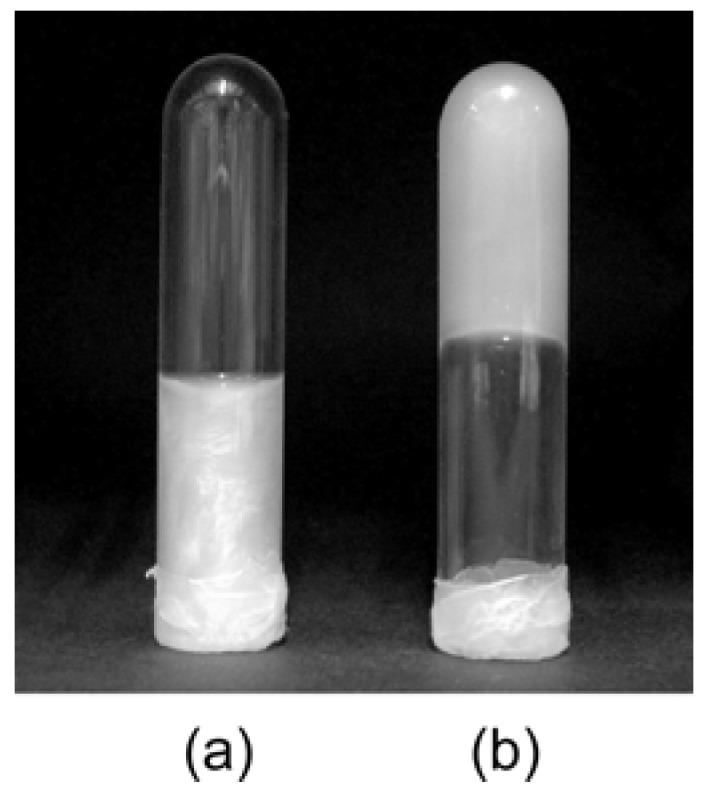
Photograph of a niobate nanosheet colloid (a) before and (b) after gelation induced by addition of H_2_SO_4_ [158]. Reprinted with permission from Ref. [158]. Copyright 2003 American Chemical Society.

**Figure 9:**
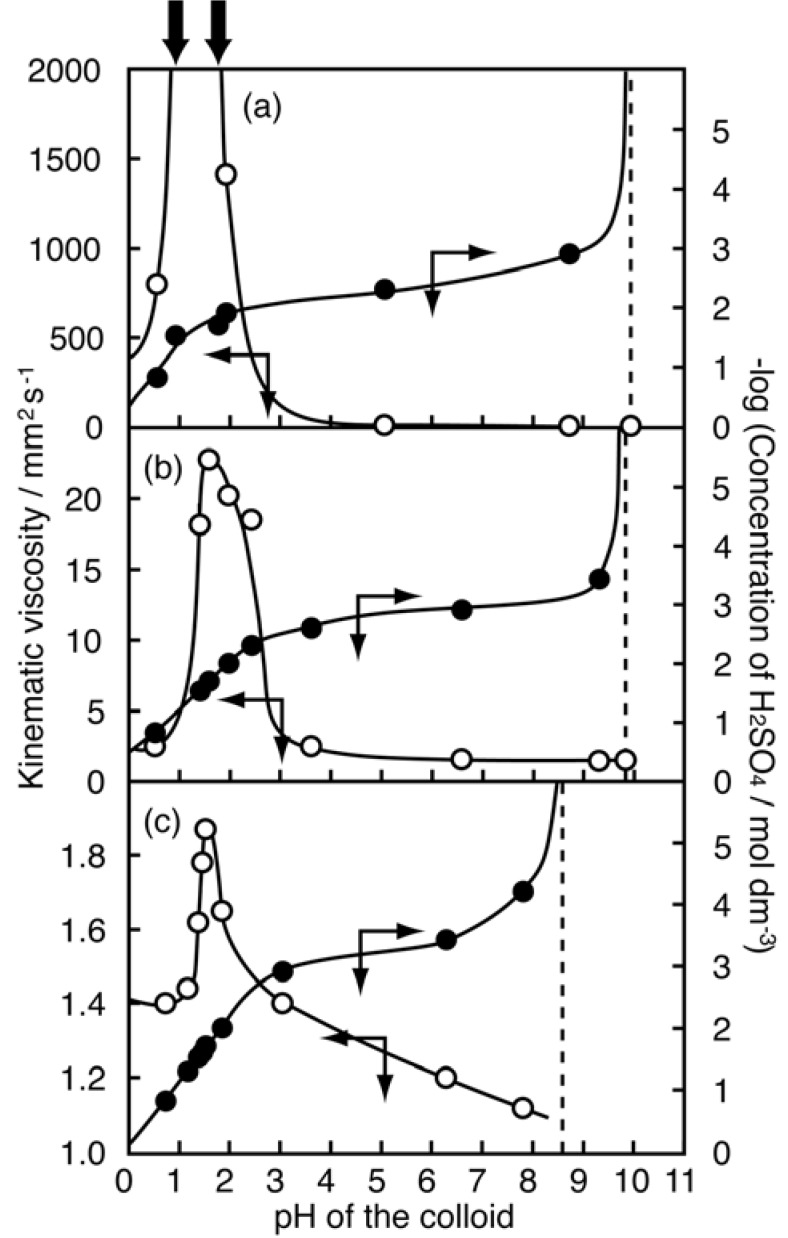
Variations in kinematic viscosity of the niobate nanosheet colloids with pH (open circles), and titration curves of the colloids (relationship between pH and concentration of H_2_SO_4_ added) (filled circles) [158]. Concentration of the colloids are (a) 7.3, (b) 2.4, and (c) 0.8 g L^–1^ of [Nb_6_O_17_]^4–^, respectively. For the colloid with 7.3 g L^–1^ of [Nb_6_O_17_]^4–^, viscosity is too high to be measured at pHs indicated by the arrows on the top. The dashed lines show pHs where [SO_4_^2–^] = 0. Reprinted with permission from Ref. [158]. Copyright 2003 American Chemical Society.

### 5.4. pH-induced gelation

We have discovered that the colloid of layered niobate K_4_Nb_6_O_17_ nanosheets exhibits pH-induced gelation that had not been reported in previous studies of nanosheet colloids [157,158]. Although the as-prepared niobate nanosheet colloid (pH 10) is a fluid sol, it becomes viscous with the addition of H_2_SO_4_ or HCl to be gelled at around pH 1.5, when the concentration of the niobate is enough high (~10 g L^–1^). The photograph and pH–viscosity relationship of the gelled sample are shown in Figure 8 and Figure 9, respectively. Optical microscope observations reveal that the orientationally ordered niobate nanosheets present in the colloid before gelation are gathered to form domains with the size of submillimeters, indicating that the gels are hierarchically constructed.

## 6. Photochemical Functions of the Niobate Nanosheet Colloids

Layered niobates have been known as semiconductor photocatalysts, and actively investigated the photocatalysis for water splitting for more than 20 years [159,160,161,162,163,164,165,166,167]. Among the investigations, exfoliated niobate nanosheets have often been utilized as building blocks of nanostructurally designed photocatalysts; for example, high-surface-area materials are obtained by exfoliation–reconstruction of layered niobates [168,169]. However, the niobate nanosheet colloids in as-prepared states have scarcely been investigated because such colloids contain organoammonium cations (typically tetrabutylammonium (TBA^+^) ions), which are introduced to the system as the exfoliating reagent and present as counter cations of the negatively charged niobate nanosheets [104,170,171,172]. Since the organic cations can be sacrificially decomposed through oxidation by the positive holes photogenerated in the semiconducting niobate layers upon photoirradiation to complicate the net reaction, the as-prepared nanosheet colloids accompanied by the organic species have not been welcomed as photocatalytic systems.

However, Osterloh and coworkers have recently studied photocatalysis of the as-prepared colloidal niobate nanosheets [173,174,175,176]. Nanosheet colloids of layered niobates HCa_2_Nb_3_O_10_ and K_4_Nb_6_O_17_ were found to photocatalytically generate hydrogen from water. An interesting finding is that whether or not the organic exfoliating reagent works as a sacrificial donor in the photocatalytic reaction is dependent on the species; TBA^+^ cations do not participate the photocatalysis although propylammonium ions enhance the photocatalysis. Both of the systems exhibit enhanced photocatalytic activities after deposition of Pt as a cocatalyst, as in the case of TiO_2_ photocatalysis.

Because the photocatalytic activity of the niobates can be utilized for photochemical reactions other than the hydrogen evolution, photochemical application of the niobate nanosheet colloids is not limited to the water splitting [177,178,179,180,181,182,183,184]. We have paid attention on photochemical function of the clay–niobate colloid, which has the phase-separated domains of clay and niobate nanosheets and exhibits selective adsorption of organic cations onto the clay nanosheets as described above [114]. The phase-separated colloidal structure means spatial separation of the clay and niobate domains with maintenance of the mobility of nanosheets. Thus, we can introduce spatially separated two functional moieties into the colloids: photocatalytically active niobate nanosheets and functional organic species (such as dyes) selectively adsorbed on the clay nanosheets.

**Figure 10:**
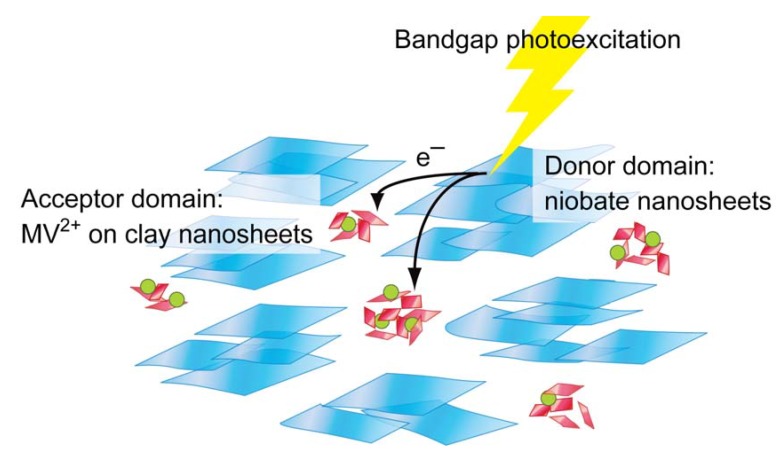
Schematic representation of the photoinduced electron transfer in the clay–niobate nanosheet colloids added by methylviologen (MV^2+^) species as electron acceptor [186]. Reprinted with permission from Ref. [186]. Copyright 2009 American Chemical Society.

We have investigated photoinduced electron transfer in the clay–niobate nanosheet colloids by assigning the two nanosheet domains to electron donating and accepting moieties [185,186]. The niobate nanosheets are used as electron donors and methylviologen (MV^2+^) cations the latter which are adsorbed on the clay nanosheets as electron acceptors. Figure 10 schematically shows the photochemical event in this system.

**Figure 11:**
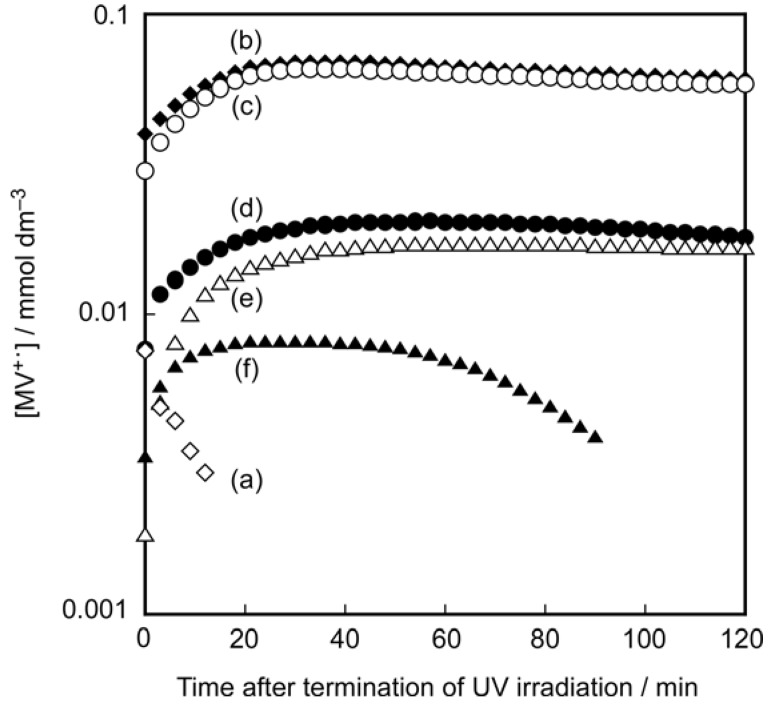
Time courses of the concentration of MV^+•^ species observed after termination of UV-irradiation in the clay–niobate colloids added by MV^2+^ with composition of [clay] = 1 (a, open diamonds), 5 (b, filled diamonds), 10 (c, open circles), 20 (d, filled circles), 25 (e, open triangles), and 30 (f, filled triangles) g L^–1^, [niobate] = 1 g L^–1^ and [MV^2+^] = 0.1 mmol L^–1^ [186]. Reprinted with permission from Ref. [186]. Copyright 2009 American Chemical Society.

The colloid causes electron transfer from the semiconducting nanosheets to the MV^2+^ acceptors to form methylviologen radical cations (MV^•+^) upon band-gap excitation of the niobate nanosheets with UV-irradiation under a nitrogen atmosphere, which is spectroscopically monitored by the formation of MV^•+^ cations. The photochemical behavior of this system is characterized by controllability of both the yield and stability of the photoproduct by the clay content. Figure 11 shows the amount of MV^•+^ cations in the colloids with various clay contents and their time-dependent decay after termination of the irradiation. The yield (maximum conversion) and lifetime of MV^•+^ vary in the range of 8 to 70% and 10 min to 40 h, respectively, depending on the clay content, indicating that rather efficient and very stable photoinduced charge-separation is achieved under the optimum conditions. Rheological measurements indicate that the coexistence of clay nanosheets increases the viscosity of the colloid, and that the largest yield and lifetime of the MV^•+^ species are obtained at a moderate viscosity. Thus, the efficient and stable photoinduced electron transfer occurs under appropriately mobile conditions for the niobate and clay nanosheets.

## 7. Concluding Remarks

We have reviewed new aspects of inorganic nanosheets as the component of functional anisotropic colloids. Permanent liquid crystal phases with optical anisotropy have been reported for nanosheet colloids and their phase behaviors are basically consistent with the theoretical prediction based on the excluded-volume effect. External forces such as magnetic, electric, and shear forces are effective to cause temporal orientation of nanosheets or to obtain a macroscopically aligned single domain of the liquid crystal phases. Due to complicated interactions among the highly anisotropic particles, the nanosheet colloids undergo intriguing sol–gel transitions. Due to the distinctive features, the nanosheet liquid crystals will find versatile applications for anisotropic reaction media, and templates for ordered nanostructures, electric and optical devices, photofunctional materials.
